# HLA Profiles of Patients With Chronic Kidney Disease in Central South Africa

**DOI:** 10.1155/ijne/5519442

**Published:** 2026-05-08

**Authors:** Walter Janse van Rensburg, Kirsten Lange, Feziwe Bisiwe, Jean Kloppers

**Affiliations:** ^1^ Human Molecular Biology Unit, School of Biomedical Sciences, University of the Free State, Bloemfontein, Free State, South Africa, ufs.ac.za; ^2^ Nephrology Clinical Unit, Department of Internal Medicine, School of Clinical Medicine, University of the Free State, Bloemfontein, Free State, South Africa, ufs.ac.za; ^3^ Department of Haematology and Cell Biology, School of Pathology, University of the Free State, Bloemfontein, Free State, South Africa, ufs.ac.za; ^4^ National Health Laboratory Service, Universitas Academic Unit, Bloemfontein, Free State, South Africa, nhls.ac.za

**Keywords:** chronic kidney disease, human leucocyte antigen, South Africa

## Abstract

**Background:**

The human leucocyte antigen (HLA) complex has been associated with the susceptibility of several hundred human diseases, increasingly so with kidney disease. HLA data are under‐represented in South Africa, and thus, their contribution to disease characterisation remains poorly understood. Therefore, there is a need for HLA typing studies among high‐risk disease populations in our region.

**Methods:**

We recruited a total of 100 participants with biopsy‐proven chronic kidney disease (CKD) attending a nephrology clinic in central South Africa. Exploratory analyses of demographic, clinical and serological characteristics were performed. High‐resolution HLA typing was conducted using DNA microarray technology.

**Results:**

Among 100 participants, a large proportion had early‐stage CKD based on glomerular filtration rate (GFR) category (CKD stage 1 and 2: 64%), yet many were classified as being at high or very high risk of disease progression to end‐stage kidney disease (ESKD) according to the ‘Kidney Disease: Improving Global Outcomes’ (KDIGO) criteria. This risk was predominantly driven by severely increased proteinuria (proteinuria category A3: 41.8%) rather than reduced estimated GFR (eGFR). Hypertension was the most common comorbidity/complication, and roughly a fifth of the cohort were HIV positive. The most prevalent autoimmune serology was antinuclear antibody (ANA) positivity, occurring in the context of a high frequency of lupus nephritis (LN) in this cohort. We observed the following recurring set of HLA alleles in this CKD cohort, alongside specific clinical and/or serological features: HLA‐A∗23:01; HLA‐A∗68:02; HLA‐B∗15:10; HLA‐B∗44:03; HLA‐DRB1∗03:01; HLADQB1∗02:01; HLA‐DQB1∗02:02 and/or HLA‐DPB1∗04:01.

**Conclusion:**

Our findings suggest that specific HLA alleles may be associated with CKD susceptibility and/or the increased risk of disease progression in this regional cohort, warranting further investigation into specific CKD‐related immunological and molecular mechanisms to establish causality in larger studies. Furthermore, by increasing our global representation in population‐specific reference panels, we can improve our understanding of South Africa’s extensive genetic diversity.

## 1. Introduction

The human leucocyte antigen (HLA) complex is a multi‐gene system that plays a crucial role in regulating innate and adaptive immune responses. This region is located on the short arm (p21.3) of chromosome 6, spans a length of 3600 kilobase pairs (kb) and is composed of three classes, namely, HLA class I (encodes classical A, B and C molecules), class II (encodes classical DR, DQ and DP molecules) and class III (does not encode HLA molecules and is not part of routine HLA typing) [1]. The HLA genes are the most polymorphic genes in the human genome and currently comprise a total of 42,996 alleles [2], with an ever‐increasing number of alleles constantly being identified. Due to the role HLA plays in the immune system, this gene region is subject to various degrees of disease susceptibility and has already been associated with several hundred human diseases [3, 4].

Different HLA markers are found to be more prevalent in certain ethnic populations, wherein those of African descent are considered to exhibit the most polymorphism [5]. The high genetic diversity observed in African populations is not well understood, and therefore, limits our understanding of susceptibility to diseases [6, 7]. HLA typing is a valuable tool used, not only in identifying potential organ donors but also in the prediction of disease progression [8]. However, HLA typing data, especially of high resolution, are under‐represented in the South African population due to a limited number of studies conducted [9].

Although HLA typing currently has its importance rooted in the field of transplant medicine, four‐digit typing data may be very useful in the context of research [10], as different disease diagnoses and drug hypersensitivity associations are sometimes only detectable at allele level [11]. Subsequently, there is a considerable amount of evidence that now supports the presence of HLA genotypic associations in kidney diseases, beyond transplantation [12]. In effect, the HLA region has been shown to be closely related to several diseases of the kidney, as well as to decreased kidney functioning [12, 13], though, a true disease association is only possible when a detailed clinical phenotype profile is considered with the appropriate high‐resolution HLA genotype [12].

According to the ‘Kidney Disease: Improving Global Outcomes (KDIGO)’ Guidelines, chronic kidney disease (CKD) is defined as abnormal kidney structure or abnormal kidney function for 3 months or more with implications for health [14]. CKD may be classified based on the proteinuria category (indicating kidney damage), the eGFR category (indicating kidney function) and the specific cause/aetiology of the disease [14]. Accurately identifying the cause of CKD is essential in guiding management and predicting prognosis and can sometimes only be possible with the use of a kidney biopsy [15].

The prevalence of CKD has been reported to be about three times higher in African countries than in higher‐income regions [16]. Hypertension and diabetes mellitus are the most common causes of CKD worldwide, and the risk is expected to increase with age. However, young adults appear to be disproportionately affected in sub‐Saharan Africa. Furthermore, in addition to hypertension and diabetes mellitus, human immunodeficiency virus (HIV) was found to be among the most common causes of end‐stage kidney disease (ESKD) in the central South African population [17]. Nonetheless, there is still a gap in knowledge regarding the prevalence of the different forms of CKD, especially in South Africa as many patients present late at the stage where the kidneys are small and not amenable to biopsy [18]. Due to this gap, we believe that the potential underlying immunological risk factors may also be under‐reported in this patient cohort.

According to one of the only two formal HLA studies conducted in a central South African population [8, 19], kidney failure was one of the most reported conditions in this region. Furthermore, a specific allele group, namely, HLA‐B∗44, was detected at a higher frequency among the kidney failure population [8]. Thus, with this study, we aimed to explore the high‐resolution HLA profiles of patients with CKD at the Universitas Academic Hospital Nephrology Clinic, in Bloemfontein, Free State, South Africa.

## 2. Materials and Methods

### 2.1. Study Design and Participants

We performed an analytical observational cross‐sectional study at the Human Molecular Biology Unit in collaboration with the Universitas Academic Hospital Nephrology Division, at the Faculty of Health Sciences, University of the Free State, South Africa. From January to June 2022, participants aged ≥ 18 years who were diagnosed with biopsy‐proven CKD were recruited from the nephrology clinic at Universitas Academic Hospital in Bloemfontein, Free State. Biopsies formed part of the inclusion criteria to accommodate a well‐defined diagnosis for the accuracy of further genotypic studies. Participants who had received recent blood transfusions and/or kidney transplant recipients were excluded, due to the potential contamination of recipient blood samples with donor DNA.

### 2.2. Procedures

Demographic and clinical information were obtained from the patients’ electronic and paper‐based records and included age, sex, self‐reported ethnicity, cause and complications of CKD, eGFR, proteinuria results and serology data. CKD was classified based on the CKD classification criteria (GFR category, G1‐G5; proteinuria/albuminuria category, A1–A3; and the specific cause) as defined in the KDIGO guidelines [14]. These classification criteria were used to assess risk of disease progression throughout the study (Table 1). Comorbidities and medication history was obtained from the medical records. The date of the kidney biopsy was used as the time of diagnosis, and the study included participants who were diagnosed with kidney disease between 2004 and 2022.

**TABLE 1:** Description and ranges for GFR and proteinuria categories, as well as predictive risk of CKD outcome (copied and modified from KDIGO, 2024) [14].

Prognosis of CKD by GFR and proteinuria categories				Proteinuria categories (g/mmol)		
				A1	A2	A3
				Normal to mildly increased	Moderately increased	Severely increased (including nephrotic syndrome)
				< 0.015	0.015–0.050	> 0.050 (> 0.350 nephrotic)
GFR categories (mL/min/1.73 m^2^)	G1	Normal to high	≥ 90			
	G2	Mildly decreased	60–89			
	G3a	Mildly to moderately decreased	45–59			
	G3b	Moderately to severely decreased	30–44			
	G4	Severely decreased	15–29			
	G5	Kidney failure	< 15			

*Note:* Green: low risk (if no other markers of kidney disease, no CKD); yellow: moderately increased risk; orange high risk; red, very high risk.

**FIGURE 1:**
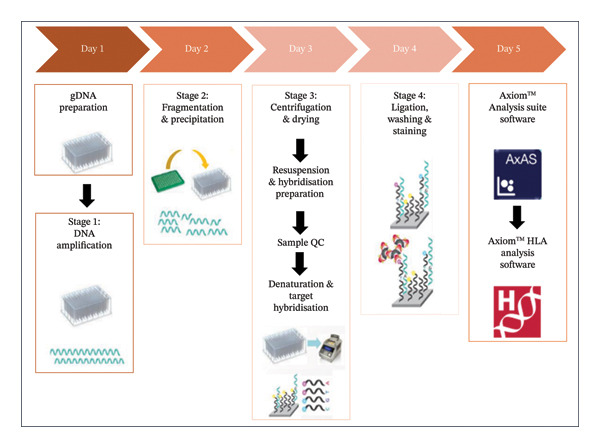
An overview of the HLA genotyping process using the Axiom microarray technology (images used from Thermo Fischer Scientific).

One ethylenediaminetetraacetic acid (EDTA)–containing tube (BD Vacutainer, reference number: 368,861, Becton Dickson, South Africa) of venous blood was collected from each study participant by a registered healthcare practitioner during the participants’ routine visits to the clinic. DNA was extracted from whole blood collected from each study participant using the Quick‐DNA Miniprep Kit (Zymo Research, USA) as per the manufacturer’s instructions. DNA concentrations ([DNA]) were determined using the QuickDrop Spectrophotometer (Molecular Devices, USA). High‐resolution HLA typing of class I (A, B and C) and class II alleles (DR, DQ and DP) was performed for each study sample, using the Axiom Precision Medicine Diversity Array (PMDA) Kit (96‐format) on the GeneTitan Multi‐channel (MC) microarray instrument according to the manufacturer’s instructions (Thermo Fisher Scientific, USA). The high‐resolution HLA typing procedure involved three main steps, namely, genomic DNA preparation, manual target preparation of the samples and finally, automated array plate processing. An overview of the genotyping process is shown in Figure 1.

### 2.3. Data Analysis

Formal statistical comparisons between subgroups were not performed due to limited subgroup sizes and the exploratory aim of the study. Instead, allele frequencies were descriptively compared across clinically relevant categories to identify patterns warranting further investigation in larger cohorts.

High‐resolution (4‐digit) HLA typing data were analysed to assess allele and haplotype frequencies, Hardy–Weinberg equilibrium (HWE) proportions and linkage disequilibrium. The HLA frequency data were placed into a single Microsoft Excel spreadsheet for analysis. The class I and II allele frequencies across the total study population were determined using Microsoft Excel functions, specifically the direct counting method (total number of copies of the allele divided by 2n) [8, 20]. The Arlequin v3.5.2 software [21] was used to obtain haplotype frequency data, based on the expectation‐maximization (EM) algorithm of maximum likelihood with at least 10,000 permutations [22, 23]. Deviations from HWE were analysed based on a chi‐square test [24]. Pairwise global linkage disequilibrium was calculated for the class I and class II loci pairs to determine the non‐random association of HLA alleles at different loci.

To describe patterns between HLA types and various demographic, clinical and serological markers, we compared HLA frequency data across demographic characteristics of interest and the following comparator groups: high risk (*n* = 27) versus low risk (*n* = 20) of disease progression; CKD stage 3A‐5 (*n* = 36) versus CKD stage 1–2 (*n* = 64); nephrotic‐range proteinuria (*n* = 13) versus normal to mildly increased proteinuria (*n* = 23); hypertensive (*n* = 62) versus non‐hypertensive (*n* = 38); HIV positive (*n* = 18) versus HIV negative (*n* = 74); presence (*n* = 43) versus absence (*n* = 55) of ANA autoantibodies. For each comparison group, only the three most frequent HLA alleles were considered. Accordingly, the term *prevalent* refers to alleles that ranked among the three most frequent within a given group. As an additional point of comparison between region‐specific HLA data, and to meet our study objective regarding the HLA‐B∗44 allele group, the low‐resolution allele‐group frequencies (2‐digit) were compared with that from the study by Janse van Rensburg et al. (2021) [8], which served as a suitable control population of healthy donors from the same central South African region (*n* = 406). The allele‐group data were also compared with a reference population of kidney failure patients (*n* = 237) from the above‐mentioned study [8].

## 3. Results

### 3.1. Demographic Characteristics

The study population consisted of a total of 100 participants (*n* = 100) histologically diagnosed with various CKDs, of which 58% (*n* = 58/100) were female and 42% (*n* = 42/100) were male. Participants were between the ages of 20 and 87 years, with a median age of 35 years (IQR = 29–46.3 years). Most of the study population consisted of individuals of African descent (84%, *n* = 84/100), followed by those of European descent (8%, *n* = 8/100), mixed ancestry (6%, *n* = 6/100) and Asian/Indian descent (2%, *n* = 2/100) (Table 2).

**TABLE 2:** Demographic characteristics of the total CKD study population.

**Demographic distribution of the CKD population (*N* = 100)**			
	**Male**	**Female**	**Total**

Number of participants (*n*)	42	58	100
Age (years)			
Median (IQR)	33 (27.3–42)	37 (30–49.8)	35 (29–46.3)
Ethnicity, *n* (%)			
African descent	36 (85.7)	48 (82.8)	84 (84.0)
European descent	2 (4.8)	6 (10.3)	8 (8.0)
Mixed ancestry	2 (4.8)	4 (6.9)	6 (6.0)
Asian/Indian descent	2 (4.8)	0 (0.0)	2 (2.0)

Abbreviations: IQR, interquartile range; SD, standard deviation.

### 3.2. Clinical Characteristics

CKD stage distribution by eGFR and proteinuria categories are shown in Tables 3 and 4, respectively. Although most individuals in the cohort were classified as having early‐stage CKD (stage 1: 52%; stage 2: 12%), reflected by the overall mean eGFR (79.9 mL/min/1.73 m^2^) indicating only mildly reduced kidney function, the majority had moderately to severely increased proteinuria, indicative of underlying kidney injury. When these parameters were considered jointly, the predicted risk of CKD progression (Table 5) showed that most participants fell into the high or very high KDIGO risk categories for progression to ESKD [14], driven predominantly by the high prevalence of severely increased proteinuria. When risk of disease progression was analysed among sub‐groups, the young African‐descent population (median age: 32 years) had a numerically higher risk of disease progression compared with the older, non‐African descent population (median age: 48 years).

**TABLE 3:** Distribution of CKD stages by GFR categories in the total population (*N* = 100).

CKD stage, (eGFR, ml/min/1.73 m^2^) [14]	Number of participants, *n* (%)	Level of kidney function [14]
Stage 1 (≥ 90)	52 (52)	Normal to high
Stage 2 (60–89)	12 (12)	Mildly decreased
Stage 3A (45–59)	8 (8)	Mild to moderately decreased
Stage 3B (30–44)	16 (16)	Moderately to severely
Stage 4 (15–29)	5 (5)	Severely decreased
Stage 5: ESKD (< 15)	7 (7)	Kidney failure
Mean eGFR of total cohort	79.9 mL/min/1.73 m^2^	

**TABLE 4:** Distribution of proteinuria categories in the total population (*N* = 98)^∗^.

Proteinuria category (g/mmol) [14]	Number of participants, *n* (%)	Degree of kidney damage [14]
A1 (< 0.015)	23 (23.5)	Normal to mildly increased
A2 (0.015–0.050)	34 (34.7)	Moderately increased
A3 (> 0.050)	41 (41.8)	Severely increased
*>* *0.050–0.350*	*28 (28.6)*	
*>* *0.350 (nephrotic)*	*13 (13.3%)*	
Mean proteinuria of total cohort	0.2 g/mmol	

*Note:* The italic values are used to subclassify the A3 group.

^∗^Two participants with unknown proteinuria results were excluded from this specific analysis.

**TABLE 5:** Risk of disease progression by GFR and proteinuria categories in the total population (*N* = 98)^∗^.

Prognosis [14]	Number of participants, *n* (%)
Low risk	20 (20.4)
Moderately increased risk	21 (21.4)
High risk	30 (30.6)
Very high risk	27 (27.6)

^∗^The two participants with unknown proteinuria results were excluded from this specific analysis.

The most common complication of CKD in the total study population was hypertension (62% *n* = 62/100), followed by anaemia (24%, *n* = 24/100). Only 8 of the 100 participants did not have a documented HIV status. Nearly 20% (19.6%, *n* = 18/92) of the participants who had a documented HIV status were HIV positive, and the rest (80.4%, *n* = 74/92) were HIV negative.

The cause of CKD was established based on the primary disease according to the histological diagnosis, as shown in Table 6. We found that LN, FSGS, MPGN, MCD and MN were the five most prevalent histological diagnoses among biopsied participants in this region, with LN accounting for a notable 38% of the total CKD population (Lange et al., 2023) [25]. Previously performed serology was obtained from the NHLS records to further aid assessment of CKD cause (Table 7) and showed that autoimmunity was prominent in this study.

**TABLE 6:** Chronic kidney diseases histologically grouped according to the primary disease (*N* = 100).

Distribution of the total CKD study population according to primary histological disease groups		
Group number	Primary histological disease group	Number of participants, *n*
1	Lupus nephritis (LN)	38
2	Focal segmental glomerulosclerosis (FSGS)	14
3	Membranoproliferative glomerulonephritis (MPGN)	12
4	Minimal change disease (MCD)	9
5	Membranous nephropathy (MN)	8
6	Hypertensive (HPT) kidney disease	5
7	ANCA‐associated vasculitis (AAV)	3
8	HIV‐associated kidney disease	3
9	IgA nephropathy (IgAN)	2
10	Tubulointerstitial nephritis (TIN)	2
11	C1q glomerulopathy (C1qG)	1
12	Microcystic tubular dilatation/pyelonephritis	1
13	Drug‐induced podocytopathy/acute tubular necrosis (ATN)	1
14	Post‐infectious glomerulonephritis	1

**TABLE 7:** Distribution of the main serological results across the total CKD study population^∗^.

Serology results of the total CKD study population	
Serological finding	Participants with positive results (%, *n*)
HIV	19.6% (*n* = 18/92)
Hepatitis B	0% (*n* = 0/89)
Hepatitis C	1.1% (*n* = 1/89)
ANA	42.9% (*n* = 42/98)
Anti‐RNP	69.7% (*n* = 23/33)
Anti‐dsDNA	54.6% (*n* = 18/33)
Anti‐Sm	54.6% (*n* = 18/33)
Anti‐Ro/SSA	30.3% (*n* = 10/33)
Anti‐Scl‐70	12.1% (*n* = 4/33)
aPL	41.5% (*n* = 17/41)
ANCA	8.5% (*n* = 7/82)
RF	15.4% (*n* = 8/52)
Anti‐CCP	*n* = 6^∗^
Anti‐GBM	0% (*n* = 0/34)
Anti‐PLA2R	*n* = 2^∗^

^∗^These autoantibodies were part of the additional serology results available, and thus only the positive results were recorded.

### 3.3. HLA Typing Results

A total of 11 major HLA class I and II loci were genotyped and included the following loci: HLA‐A; ‐B; ‐C; ‐DPA1; ‐DPB1; ‐DQA1; ‐DQB1; ‐DRB1; ‐DRB3; ‐ DRB4 and ‐DRB5. Only 8 of the 11 loci had sufficient different alleles per loci (*n* ≥ 5 alleles per loci) to allow for adequate descriptive data analysis to be performed. Of the total CKD population (*N* = 100), 179 different possible HLA class I and II alleles were identified, of which the HLA‐B locus exhibited the highest degree of genetic diversity (*n* = 45).

All the HLA allele subsets in our study deviated from the expected HWE proportions (*p* < 0.05), and consequently the HLA haplotype frequencies could not be determined. Strong linkage disequilibrium was determined within the HLA class I loci and class II loci, as well as between the class I and class II loci. The linkage disequilibrium was found to be *p* < 0.05 in the total study population.

The three most frequent allele‐groups and specific alleles across the eight most prevalent HLA loci are summarised in Table 8.

**TABLE 8:** The most common HLA allele (4‐digits) and allele‐group frequencies (2‐digit) in the total CKD study population.

**The three most prevalent HLA allele and allele-group frequencies across the total CKD study population (*N* = 100)**				
	**Most prevalent 4-digit HLA alleles (%)**	**HLA gene frequencies (4-digit)**	**Most prevalent 2-digit HLA Allele-groups (%)**	**HLA gene frequencies (2-digit)**

HLA‐A	HLA‐A∗23:01 (11%)	33	HLA‐A∗02 (18%)	19
	HLA‐A∗68:02 (9%)		HLA‐A∗68 (13.5%)	
	HLA‐A∗30:01 (7.5%)		HLA‐A∗23 (11%)	

HLA‐B	HLA‐B∗58:02 (9%)	45	HLA‐B∗15 (17.5%)	24
	HLA‐B∗44:03 (8.5%)		HLA‐B∗58 (12%)	
	HLA‐B∗15:10 (7.5%)		HLA‐B∗44 (11%)	

HLA‐C	HLA‐C∗04:01 (18%)	22	HLA‐C∗07 (24%)	15
	HLA‐C∗06:02;		HLA‐C∗04 (18%)	
	HLA‐C∗07:01 (13%)		HLA‐C∗06 (13%)	
	HLA‐C∗02:02 (11%)			

HLA‐DPA1	HLA‐DPA1∗01:03 (30.5%)	7	n/a	n/a
	HLA‐DPA1∗02:01 (30%)			
	HLA‐DPA1∗02:02 (24%)			

HLA‐DPB1	HLA‐DPB1∗01:01 (33%)	16	n/a	n/a
	HLA‐DPB1∗04:02 (17.5%)			
	HLA‐DPB1∗04:01 (10.5%)			

HLA‐DQA1	HLA‐DQA1∗01:02 (30%)	8	n/a	n/a
	HLA‐DQA1∗03:01 (18%)			
	HLA‐DQA1∗05:01 (17.5%)			

HLA‐DQB1	HLA‐DQB1∗06:02 (24.5%)	16	HLA‐DQB1∗06 (34.5%)	5
	HLA‐DQB1∗02:01 (11.5%)		HLA‐DQB1∗03 (25.5%)	
	HLA‐DQB1∗02:02 (10.5%)		HLA‐DQB1∗02 (22%)	

HLA‐DRB1	HLA‐DRB1∗07:01 (13.5%)	32	HLA‐DRB1∗15 (17%)	13
	HLA‐DRB1∗15:03 (12.5%)		HLA‐DRB1∗04 (14%)	
	HLA‐DRB1∗03:01 (8%)		HLA‐DRB1∗03; HLA‐DRB1∗07 (13.5%)	

HLA alleles of interest which were prevalent in our study, but not in healthy, multi‐ethnic South Africans were: HLA‐A∗23:01; HLA‐A∗68:02; HLA‐B∗44:03; HLA‐B∗15:10; HLA‐C∗02:02; HLA‐DRB1∗03:01; HLA‐DQB1∗02:01; HLA‐DQB1∗02:02; HLA‐DPB1∗04:02; HLA‐DPB1∗04:01. The HLA‐DPA1 and HLA‐DQA1 loci were not studied by Lange et al. [25] or Tshabalala et al. [26] and could thus not be compared. In contrast, the HLA alleles which were prevalent in our study, as well as healthy South Africans were as follows: HLA‐A∗30:01, HLA‐B∗58:02, HLA‐C∗04:01, HLA‐C∗06:02, HLA‐C∗07:01, HLA‐DRB1∗07:01, HLA‐DRB1∗15:03, HLA‐DQB1∗06:02 and HLA‐DPB1∗01:01.

The HLA allele groups that were prevalent in our study but not in the healthy South African donors were: HLA‐A∗23; HLA‐B∗58; HLA‐DRB1∗04 and HLA‐DRB1∗07. The HLA allele‐groups that were prevalent in our study as well as in the kidney failure reference population were: HLA‐A∗02; HLA‐A∗68; HLA‐A∗23; HLA‐B∗44; HLA‐B∗58; HLA‐C∗04; HLA‐C∗06; HLA‐C∗07 and HLA‐DRB1∗03.

### 3.4. HLA Typing Results in Relation to Observed Clinical/Serological Characteristics

To explore patterns between HLA alleles of interest and specific clinical and serological characteristics, we considered the three most prevalent HLA alleles across the clinical and serological characteristic groups included in this study.

HLA‐A∗23:01 was the most prevalent HLA‐A allele in the total CKD study cohort (11%) and was common in the LN (11.8%), FSGS (14.3%) and MPGN (12.5%) disease groups. The HLA‐A∗68:02 allele was among the most prevalent HLA‐A alleles in the CKD cohort as well as in all the main disease groups (LN, 11.8%; MPGN, 12.5%; MCD, 11.1%; MN, 12.5%), except for FSGS. The HLA‐B∗44:03 allele was the second most prevalent HLA‐B allele in the total CKD population but was the most common in the LN cohort (11.8%). This allele was also prevalent in the MPGN (8.3%) and MCD (16.7%) groups, but not in the FSGS and MN groups. The HLA‐B∗15:10 allele was prevalent across all the main disease groups (FSGS, 7.1%; MPGN, 12.5%; MCD, 11.1%; MN, 12.5%), except for LN. HLA‐C∗02:02 was prevalent in the FSGS (21.4%) and MPGN (12.5%) disease groups. The HLA‐DRB1∗03:01 allele was prevalent in all the disease groups (LN, 6.6%; FSGS, 14.3%; MPGN, 12.5%; MCD, 11.1%), except for MN. The HLA‐DQB1∗02:01 and HLA‐DQB1∗02:02 alleles were of interest in the total CKD population, though, the HLA‐DQB1∗02:01 allele was more prevalent in the FSGS (17.9%) and MCD (16.7%) disease groups, whereas the HLA‐DQB1∗02:02 allele was more prevalent in the LN (10.5%) and MN (18.8%) disease groups. The HLA‐DPB1∗04:01 and HLA‐DPB1∗04:02 alleles were both prevalent in the total CKD population, though, only the HLA‐DPB1∗04:02 allele was prevalent in the main disease groups (LN, 17.1%; FSGS, 14.3%; MPGN, 25%; MCD, 11.1%; MN, 12.5%).

The HLA‐A∗23:01 allele was only present among individuals of African descent, though, this finding may be influenced by the predominant proportion of African‐descent participants in the study. Of the study population with a very high risk of progression to ESKD, HLA‐A∗23:01 had an increase in frequency of 11.1% versus 5% in the very high‐risk group versus the low‐risk group. The relative increase in frequency of this allele between those with CKD stage 3A‐5 versus stage 1‐2 was descriptively similar (11.1% versus 10.9%); however, that between individuals with nephrotic‐range proteinuria versus normal to mildly increased proteinuria was observed at higher frequencies (19.2% versus 6.5%). Furthermore, this allele was not of interest in overall ANA positivity.

Similar frequencies of the HLA‐A∗68:02 allele were found between those with a very high risk (9.3%) and those with a low risk (10%) of disease progression, and thus, no specificity was shown to either a decrease in eGFR or severely increased proteinuria. HLA‐A∗68:02 was prevalent in both the hypertensive and HIV positive groups, as well as across all the antibody groups studied.

The HLA‐B∗15 allele group was the most common allele group in the total CKD population. The HLA‐B∗15:10 allele, among the most prevalent HLA‐B alleles, was not prevalent in the group at increased risk of progression to ESKD and was therefore not observed to be associated with decreased kidney function or severely increased proteinuria. This allele was of similar frequency among the hypertensive (7.3%) and non‐hypertensive groups (7.9%), as well as the HIV positive (11.1%) and negative (7.4%) groups. HLA‐B∗15:10 also exhibited similar frequencies among the ANA positive (7%) and negative groups (8.2%) but was only of prevalence within the anti‐dsDNA (7.9% positive, 8% negative) and anti‐Ro/SSA (10% positive, 6.3% negative) ANA subtype groups.

The HLA‐B∗44 allele group, of particular interest in our study due to its previously reported prevalence in people with kidney failure in our region [8], was found to have two specific alleles in our population, namely, the HLA‐B∗44:03 and HLAB∗44:02 alleles. The HLA‐B∗44:02 allele was specific to those of European descent, whereas the HLA‐B∗44:03 allele was specific to those of African descent. The HLA‐B∗44:03 allele was observed among those with a very high risk of progression to ESKD (7.4%), though, the relative increase in frequency was higher in the low‐risk group (10%). This allele was prevalent across CKD stage (stage 3A‐5, 6.9%; stage 1‐2, 9.4%) but not in those with nephrotic‐range proteinuria. Of note, this allele was the most prevalent in the ANA positive group and all ANA subtypes studied, except for anti‐Ro/SSA.

The HLA‐DRB1∗03:01 allele was the most prevalent HLA‐DRB1 allele in those with a high risk of progression to ESKD, with a relative increase in frequency of 11.1% in those with a very high risk compared with 5% in those with a low risk. Relatively similar frequencies of this allele were found in those with CKD stage 3A‐5 (9.7%) versus CKD stage 1‐2 (7%) but were numerically increased in those with nephrotic‐range proteinuria (15.4%) versus those with normal to mildly increased proteinuria (6.5%). HLA‐DRB1∗03:01 was more prevalent in the HIV positive group (13.9%) versus the HIV negative group (6.8%) but may have been influenced by the difference in sample size. This allele also showed no clear link to ANA positivity, as it was not prevalent in either the ANA positive or negative groups.

Among participants with a high risk of progression to ESKD, the HLA‐DQB1∗02:01 allele had a relative increase in frequency of 14.8% in the very high‐risk group versus 7.5% in the low‐risk group. Although this allele was prevalent at similar frequencies among those with CKD stage 3A‐5 (12.5%) versus those with CKD stage 1‐2 (10.9%), it was prevalent at higher observed frequencies among those with nephrotic‐range proteinuria (15.4%) versus those with normal to mildly increased proteinuria (8.7%). The HLA‐DQB1∗02:01 allele was not prevalent in either the ANA positive or negative group; however, HLA‐DQB1∗02:02 was prevalent in both the ANA positive (9.3%) and negative (10%) groups, at similar frequencies. When examining this frequency in the ANA subtype groups, we found that the HLA‐DQB1∗02:02 allele was prevalent in the anti‐Sm group (13.9% positive vs 8% negative) and in the anti‐RNP group (13% positive vs 7.9% negative).

While the HLA‐DPB1∗04:02 allele was prevalent in all disease groups, it exhibited similar frequencies across disease progression groups (very high risk, 16.7%; low risk, 15%), CKD stage groups (stage 3A‐5, 18.1%; stage 1‐2, 17.2%) and proteinuria groups (nephrotic range proteinuria, 15.4%; normal to mildly increased proteinuria, 15%). The HLA‐DPB1∗04:01 allele, which was not prevalent in disease groups, was prevalent in those with a very high risk of disease progression (13%) compared with those with a low risk (7.5%) and was specifically increased in those with CKD stage 3A‐5 (15.3%) versus those with CKD stage 1‐2 (7.8%). This allele was not prevalent in the proteinuria comparator groups. Although this allele was present at similar frequencies in the ANA positive group (8.1%) compared with the ANA negative group (10%), it was found at higher observed frequencies among all the ANA positive subtype groups, namely, anti‐dsDNA (10.5% positive vs 6% negative), anti‐Sm (13.9% positive vs 4% negative), anti‐RNP (13% positive vs 7.9% negative) and anti‐Ro/SSA (20% positive vs 4.7% negative).

The HLA alleles that were prevalent in both our study cohort and in the kidney failure reference population^8^ are summarised in Table 9, along with descriptive comparisons of their frequencies across CKD stages, proteinuria categories, risk of disease progression and the five most common histological disease groups. Due to the insufficient and imbalanced subgroup sizes, comparisons should be interpreted with caution and considered in the context of relevant allele frequencies and comparator group size.

**TABLE 9:** Observed distribution of HLA alleles of interest across risk of disease progression groups, proteinuria category groups, CKD stage and histological diagnosis.

HLA allele (allele frequency; *N* = 100)	Risk of disease progression (very high risk, *n* = 27; low risk, *n* = 20)	Proteinuria category (nephrotic‐range, *n* = 13; normal to mildly increased, *n* = 23)	CKD stage (stage 3A–5, *n* = 36; stage 1‐2, *n* = 64)	Histological diagnosis (LN, *n* = 38; FSGS, *n* = 14; MPGN, *n* = 12; MCD, *n* = 9; MN, *n* = 8)
HLA‐A∗23:01 (11%)	Very high risk: 11.1% Low risk: 5%	Nephrotic range: 19.2% Normal to mildly increased: 6.5%	Stage 3A–5: 11.1% Stage 1‐2: 10.9%	LN (11.8%)
				FSGS (14.3%)
				MPGN (12.5%)

HLA‐A∗68:02 (9%)	Very high risk: 9.3% Low risk: 10%	*No observed prevalence*	*No observed prevalence*	LN (11.8%)
				MPGN (12.5%)
				MCD (11.1%)
				MN (12.5%)

HLA‐B∗15:10 (7.5%)	No observed prevalence	*No observed prevalence*	*No observed prevalence*	FSGS (7.1%)
				MPGN (12.5%)
				MCD (11.1%)
				MN (12.5%)

HLA‐B∗44:03 (8.5%)	Very high risk: 7.4% Low risk: 10%	*No observed prevalence*	Stage 3A–5: 6.9% Stage 1‐2: 9.4%	LN (11.8%)
				MPGN (8.3%)
				MCD (16.7%)

HLA‐DRB1∗03:01 (8%)	Very high risk: 11.1% Low risk: 5%	Nephrotic range: 15.4% Normal to mildly increased: 6.5%	Stage 3A–5: 9.7% Stage 1‐2: 7%	LN (6.6%)
				FSGS (14.3%)
				MPGN (12.5%)
				MCD (11.1%)

HLA‐DQB1∗02:01 (11.5%)	Very high risk: 14.8%	Nephrotic range: 15.45	Stage 3A–5: 12.5%	FSGS (17.9%)
	Low risk: 7.5%	Normal to mildly increased: 8.7%	Stage 1‐2: 10.9%	MCD (16.7%)

HLA‐DQB1∗02:02 (10.5%)	No observed prevalence	*No observed prevalence*	No observed prevalence	LN (10.5%)
				MN (18.8%)

HLA‐DPB1∗04:01 (10.5%)	Very high risk: 13%	*No observed prevalence*	Stage 3A–5: 15.3%	*No observed prevalence*
	Low risk: 7.5%		Stage 1‐2: 7.8%	

## 4. Discussion

The highly polymorphic nature of the HLA system, particularly at the allele level, presents an inherent challenge in studies conducted in genetically diverse populations. While our cohort of 100 participants with biopsy‐proven CKD represents one of the largest high‐resolution HLA datasets from this region, subgroup analyses were based on small numbers and should be interpreted with caution. Accordingly, the allele frequency patterns described here are intended to be descriptive and hypothesis‐generating rather than definitive evidence of association. Our study population indicated a female predominance; however, the male population was found to have a younger median age (males: 33 years vs females: 37 years), which is in keeping with a central South African study wherein relatively young males (40 years) made up the largest proportion of individuals with ESKD accepted for KRT [17]. We determined that the ethnic distribution of our CKD cohort is a good representation of that in the general population, both in the Free State and in the South African population, with very similar frequencies [28]. Thus, no ethnic discrepancies were recorded across the total CKD population in our study.

Despite early‐stage CKD in a large proportion of participants, the high prevalence of severely increased proteinuria resulted in most participants being classified as high or very high risk for progression to ESKD, according to KDIGO criteria. Hypertension was the most common recorded comorbidity/complication of CKD. However, hypertension as a cause of the histological kidney manifestation was rare in our study. Similarly, HIV as a primary cause was not as prominent as we expected and contradicts the reportedly high prevalence of hypertension and HIV as causes of ESKD in central South Africa [17]. However, a decline in classic HIV‐associated nephropathy (HIVAN) has been described in the South African population, attributed to the antiretroviral therapy (ART) rollout campaign in the country [29], which may explain the low prevalence in our study. Among the five most prevalent histological diagnoses (LN, FSGS, MPGN, MCD and MN), LN was notably common, indicating that it may be one of the major causes of CKD in this region [25]. We found autoimmunity to be more prominent in our study, as opposed to the previously reported high rate of infection‐related causes [30]. This is further substantiated by the fact that only five participants in the study had no positive autoimmune serology, but this finding may be biased by the large proportion of LN participants. Consequently, the serological profile of most participants with CKD in our study displayed positive ANA serology which is in keeping with autoimmunity.

The HLA alleles deviated from the expected HWE proportions (*p* < 0.05) and were expected based on the extensive genetic diversity exhibited in the South African population. This is not well understood due to a limited number of HLA typing studies conducted and is partly due to the major challenge encountered in the classification of ethnicity in South Africa, as a result of our complex cultural diversity [8].

The HLA alleles of interest in the current study excluded those HLA alleles also present in healthy South Africans [8, 26, 27]; however, the kidney failure population previously investigated were considered [8].

Given that the HLA‐A∗23:01 allele was prevalent across various disease groups (LN, FSGS and MPGN) with different pathogeneses, we believe that a relationship may exist between this specific allele and severely increased proteinuria, resulting in a high risk of disease progression, especially among African descent individuals in our region. Similarly, HLA‐A∗68:02 was detected at relatively high frequencies throughout all the groups of comparison, but not in FSGS, which may indicate a protective effect in those with the FSGS lesion in central South Africa. However, the nature of this effect remains unclear as no specific clinical or serological markers were identified to substantiate a causative role.

The HLA‐B∗15:10 allele was prevalent across all main disease groups, except for LN, although it was detected at similar frequencies among the ANA positive and negative groups. Thus, we hypothesise that when ANA positivity is present, it is most likely because of anti‐dsDNA or anti‐Ro/SSA antibody production, leading to a kidney manifestation other than LN. The nature of the kidney manifestation is, however, unclear, as this allele was not implicated in the increased risk of disease progression and, subsequently, was not found to be linked to either decreased kidney function or severely increased proteinuria in our CKD cohort. Factors that may have confounded this relationship were also limited due to the similar frequencies of this allele found in both the hypertensive and non‐hypertensive groups as well as the HIV positive and negative groups. This may suggest that HLA‐B∗15:10 is specific to anti‐dsDNA or anti‐Ro/SSA antibody production, but likely leads to a kidney disease other than LN.

As previously mentioned, the HLA‐B∗15 allele group was not prevalent in the kidney failure reference population but was of prevalence in the healthy control population [8]. This may be explained by the fact that the HLA‐B∗15 allele group exhibited the highest allelic diversity of the HLA‐B alleles, of which only the HLA‐B∗15:10 allele emerged as the most prevalent HLA‐B∗15 allele, potentially related to disease. Thus, the HLA‐B∗15 alleles are possibly region‐specific, except for HLA‐B∗15:10, which appears to be linked to the production of anti‐dsDNA or anti‐Ro/SSA antibody production in our CKD cohort.

The HLA‐B∗44:03 allele was prevalent in the African descent population group. Based on our results and given that HLA associations in LN are more related to the autoantibody causing the disease [31], we may deduce that the HLA‐B∗44:03 allele is possibly involved in ANA production of SLE‐specific antibody subtypes. However, a relationship between this allele and a very high risk of progression to ESKD was observed, specifically in the form of decreased eGFR. Thus, we hypothesise that HLA‐B∗44:03 is potentially linked to a greater risk of developing LN in those with SLE. This may be due to its prevalence among the ANA positive group with decreased kidney function, indicating that the kidney is a target organ.

Interestingly, we found no observed prevalence of HLA‐B∗44:03 in the proteinuria category. We hypothesise that the absence of HLA‐B∗44:03‐associated proteinuria may allude to the fact that proteinuria and eGFR are related but non‐equivalent clinical phenotypes in LN. In LN, it was previously reported that proteinuria is primarily a sign of active glomerular immune complex‐mediated injury, whereas long‐term changes in eGFR are associated with tubulointerstitial inflammation and chronic structural damage, which can progress independently of glomerular protein leakage [32, 33]. Furthermore, studies reporting associations between HLA‐B∗44:03 and higher eGFR further suggest that this allele may influence baseline renal function or filtration capacity, rather than mechanisms specifically related to immune complex‐mediated glomerular injury [34]. Thus, we suggest that any effect of HLA‐B∗44:03 (an HLA class I allele) on renal symptoms may occur through immune or inflammatory pathways that do not directly influence proteinuria measurements. Consequently, we believe that the lack of association with proteinuria in our cohort does not preclude a potential role for HLA‐B∗44:03 in LN susceptibility or progression but rather suggests that any such role is likely to be phenotype‐specific and warrants further investigation.

Additionally, we suggest that the increased prevalence of the HLA‐B∗44 allele group in the reference kidney failure population may be attributed to the high prevalence of LN in the central South African CKD population, as observed in our study [25]. However, these findings require further investigation in larger cohorts, especially given that this allele group has also been reported to be of prevalence in a healthy central South African donor population [8]. Nonetheless, this does indicate that the two forms of the HLA‐B∗44 allele group identified in our cohort may exhibit different causative roles in different ethnic groups.

The HLA‐DRB1∗03:01 and HLA‐DPB1∗04:01 alleles were common among those at very high risk of progression to ESKD, likely reflecting decreased kidney function. Similarly, HLA‐DQB1∗02:01 was highly prevalent in CKD participants with a very high risk of disease progression, though potentially linked to severely increased proteinuria. Notably, the HLA‐DQB1∗02:01 allele was more frequently observed in FSGS and MCD, two major causes of nephrotic syndrome [35].

We can conclude that a recurring set of HLA alleles were observed in this CKD cohort from central South Africa: HLA‐A∗23:01, HLA‐A∗68:02, HLA‐B∗15:10, HLA‐B∗44:03, HLA‐DRB1∗03:01, HLA‐DQB1∗02:01, HLA‐DQB1∗02:02 and/or HLA‐DPB1∗04:01, alongside specific clinical and/or serological characteristics. While validation of these patterns will require haplotype‐based analyses in larger, independent cohorts, we believe that these findings provide a strong foundation for future investigations into CKD‐related immunological and molecular mechanisms.

Additionally, we hypothesise that the HLA‐A∗30:01, HLA‐B∗58:02, HLA‐C∗04:01, HLA‐C∗06:02, HLA‐C∗07:01, HLA‐DRB1∗07:01, HLA‐DRB1∗15:03, HLA‐DQB1∗06:02 and HLA‐DPB1∗01:01 alleles identified in our study may be region‐specific rather than CKD‐specific, as they have been previously reported in HLA studies of healthy South African populations [26, 27]. While our comparisons should be interpreted with caution, we believe these findings may contribute to our global representation in population‐specific reference panels and improve our understanding of the vast genetic diversity exhibited in South Africa.

## 5. Conclusion

The HLA profiles observed in our study are supportive of the fact that certain HLA alleles may be associated with CKD susceptibility and/or the increased risk of progression to ESKD in our region. It is to be noted that we do not suggest the presence of susceptibility alleles in each locus. However, we propose that this HLA profile be used as a reference to guide further investigation of these alleles in larger cohorts and subsequently aid in CKD risk assessment. Furthermore, these findings have the potential to inform early identification of certain kidney diseases and guide strategies to prevent disease progression, once causal relationships are validated in future studies.

### 5.1. Limitations

While statistical testing such as chi‐square analysis could be informative, the small subgroup sizes in this cohort limited the validity of such analyses. As such, statistical testing was deferred to avoid overinterpretation of potentially unstable estimates.

## Author Contributions

Walter Janse van Rensburg conceptualised the study, extracted the HLA data from the raw microarray data, performed data analysis, wrote parts of the manuscript and supervised the project.

Kirsten Lange performed the participant recruitment, clinical and demographic data acquisition, practical execution, data analysis and wrote the manuscript.

Feziwe Bisiwe performed the clinical oversight and interpretation and critically evaluated the manuscript.

Jean Kloppers performed data analysis and critically evaluated the manuscript.

## Funding

The study was partly funded by an NHLS Research Trust Developmental Grant (PR2222674).

## Ethics Statement

The study was conducted in accordance with the principles of the Declaration of Helsinki. All the necessary study approvals were obtained from the Health Sciences Research Ethics Committee (HSREC) of the University of the Free State (clearance number: *UFS-HSD2021/1462/2501*), the Free State Department of Health (FSDOH) (reference number: *FS_202112_005*) and the Business Manager of the National Health Laboratory Service (NHLS) at the Universitas Academic Business Unit in Bloemfontein, to review and utilise specific NHLS results. Voluntary written informed consent and a signed genetic information document were obtained from all study participants. All samples were pseudo‐anonymised to protect the identity of the participants. This study was conducted in compliance with the South African Protection of Personal Information (POPI) Act 4 of 2013.

## Conflicts of Interest

The authors declare no conflicts of interest.

## Data Availability

Data are available upon request from the authors.
